# Diagnostic and Prognostic Role of Preoperative Circulating CA 15-3, CA 125, and Beta-2 Microglobulin in Renal Cell Carcinoma

**DOI:** 10.1155/2014/689795

**Published:** 2014-02-17

**Authors:** Giuseppe Lucarelli, Pasquale Ditonno, Carlo Bettocchi, Antonio Vavallo, Monica Rutigliano, Vanessa Galleggiante, Angela Maria Vittoria Larocca, Giuseppe Castellano, Loreto Gesualdo, Giuseppe Grandaliano, Francesco Paolo Selvaggi, Michele Battaglia

**Affiliations:** ^1^Department of Emergency and Organ Transplantation-Urology, Andrology and Kidney Transplantation Unit, University of Bari, piazza G. Cesare, 11, 70124 Bari, Italy; ^2^Department of Biomedical Sciences, Section of Hygiene, University of Bari, Piazza G. Cesare 11, 70124 Bari, Italy; ^3^Department of Emergency and Organ Transplantation-Nephrology, Dialysis and Transplantation Unit, University of Bari, Piazza G. Cesare 11, 70124 Bari, Italy; ^4^Department of Medical and Surgical Sciences-Nephrology, Dialysis and Transplantation Unit, University of Foggia, Viale Pinto 1, 71100 Foggia, Italy

## Abstract

CA 15-3, CA 125 and **β**-2 microglobulin are three common tumor markers currently used for diagnosis, prognosis, assessment of therapeutic response, and/or to evaluate recurrence in breast and ovarian cancer and malignant lymphoproliferative disorders, respectively. In the present prospective study we assessed the role of these three serum proteins as biomarkers for renal cell carcinoma (RCC), as well as any association between tumor marker levels and clinical-pathological parameters. CA 15-3, CA 125, and **β**-2 microglobulin were preoperatively measured in 332 patients who underwent nephrectomy for RCC. Estimates of cancer-specific survival (CSS) was calculated according to the Kaplan-Meier method. Multivariate analysis was performed to identify the most significant variables for predicting CSS. Preoperatively, 35.2% (*n* = 117), 9.6% (*n* = 32) and 30.4% (*n* = 101) of the patients had abnormal levels of CA 15-3, CA 125 and **β**-2 microglobulin, respectively. Statistically significant differences resulted between CA 15-3, CA 125 and **β**-2 microglobulin values and tumor size, Fuhrman grade, presence of lymph node, and visceral metastases. CSS was significantly decreased for patients with high levels of CA 15-3, CA 125, and **β**-2 microglobulin (*P* < 0.0001, *P* < 0.0001, and *P* = 0.001, resp.). At multivariate analysis only age, the presence of visceral metastases, and high levels of CA 15-3 were independent adverse prognostic factors for CSS.

## 1. Introduction

Renal cell carcinoma (RCC) accounts for approximately 3% of all adult malignancies. It is estimated that in 2013, about 65,000 new cases will be diagnosed and nearly 14,000 patients will die of RCC in the United States [1]. RCC is increasingly diagnosed at an early stage in many countries, which has likely contributed to the recent leveling of RCC mortality in the United States and many European countries. Nevertheless, despite these advances in diagnosis, up to 30% of the patients have metastatic disease at the time of diagnosis, and around 20–30% of subjects undergoing surgery will suffer recurrence [2–4].

In recent years there has been a growing interest in tumor markers not only for diagnostic purposes but also to improve the predictive power of clinical and pathological factors in prognostic models. A prognostic role has been proposed for several circulating biomarkers associated with different features of cancer cell biology, but no clinically useful marker is yet available for RCC. Many proteins have been investigated [5], including carbonic anhydrase IX (CAIX) [6], hypoxia-inducible factor-1*α* (HIF1*α*) [7], vascular endothelial growth factor (VEGF) [8], and C-reactive protein (CRP) [9, 10].

CA 15-3, CA 125, and *β*-2 microglobulin are three common tumor markers currently used for diagnosis, prognosis, assessment of therapeutic response, and/or to evaluate recurrence in breast and ovarian cancer and malignant lymphoproliferative disorders, respectively. In the present prospective study we assessed the role of these three serum proteins as biomarkers for RCC, as well as any association between tumor marker levels and clinical-pathological parameters.

## 2. Material and Methods

### 2.1. Study Population

Serum CA 15-3 (normal range: 0–25 U/mL), CA 125 (normal range: 0–35 U/mL), and *β*-2 microglobulin (normal range: 0–2.6 mg/L) were preoperatively measured in a cohort of 332 consecutive patients who underwent radical or partial nephrectomy for RCC at our institution between August 2008 and February 2013 and in 113 healthy adult volunteers with no evidence of malignancy. Written informed consent to take part was given by all participants. All patients were preoperatively staged by thoracoabdominal Computed Tomography (CT) or Magnetic Resonance Imaging. Tumor staging was reassigned according to the 7th edition of the AJCC-UICC TNM classification [11]. The 2004 World Health Organization and Fuhrman classifications were used to attribute histological type and nuclear grade, respectively.

CA 15-3, CA 125, and *β*-2 microglobulin were measured in serum samples using solid phase, two-site sequential chemiluminescent assays (CLIA) that were fully processed on a LIAISON (for CA 15-3 and CA 125) or IMMULITE 2000 (for *β*-2 microglobulin) automated random access immunoassay analyzer. Patients with an estimated glomerular filtration rate (eGFR calculated using MDRD equation) <60 mL/min/1.73 m^2^and with other known causes of elevated levels of the three biomarkers were excluded from the study (namely, patients with other malignant tumors, ovarian cysts, breast and liver diseases, and pulmonary fibrosis).

### 2.2. Statistical Analysis

Statistical calculations were performed with MedCalc 9.2.0.1 (MedCalc software, Mariakerke, Belgium) and PASW 18 software (PASW 18, SPSS, Chicago, IL, USA). Comparisons of biomarkers median values between different groups were evaluated by Mann-Whitney *U* test. In the cancer-specific survival (CSS) analysis, patients still alive or lost to followup were censored, as well as patients who died from RCC-unrelated causes. Progression-free survival (PFS) was calculated from the date of surgery to the date of disease recurrence. Estimates of CSS and PFS were calculated according to the Kaplan-Meier method and compared with the log-rank test. Spearman's correlation was applied to evaluate associations between biomarkers and tumor size/grade. Univariate and multivariate analyses were performed using the Cox proportional hazards regression model to identify the most significant variables for predicting CSS and PFS. The backward selection procedure with removal criterion *P* > 0.10 based on likelihood ratio tests was performed. A *P* value < 0.05 was considered statistically significant.

## 3. Results

Detailed clinical and pathological characteristics of the patients are summarized in Table 1. Median age at diagnosis was 63 years (range: 20–85); median pathological tumor size was 4.5 cm (range: 0.4–24 cm). Preoperatively, 35.2% (*n* = 117), 9.6% (*n* = 32), and 30.4% (*n* = 101) of the patients had abnormal levels (i.e., above the upper limit of the reference range) of CA 15-3, CA 125, and *β*-2 microglobulin, respectively. Median CA 15-3, CA 125, and *β*-2 microglobulin serum levels were significantly higher in RCC patients than healthy subjects (Table 1). Statistically significant differences resulted between CA 15-3, CA 125, and *β*-2 microglobulin values and tumor size (≤7 versus >7 cm), Fuhrman grade (≤2 versus >2), presence of lymph node, and visceral metastases (Tables 2 and 3). These results were confirmed by Spearman's correlation comparing tumor size and grade, for CA 15-3, CA 125, and *β*-2 microglobulin (*r*_*s*_ = 0.36/0.21, and *r*_*s*_ = 0.20/0.21, *r*_*s*_ = 0.24/0.23, resp., all *P* values < 0.0001).

Moreover, the three markers were intercorrelated, CA 15-3 values being correlated both with CA 125 values (*r*_*s*_ = 0.28; *P* < 0.0001) and with *β*-2 microglobulin values (*r*_*s*_ = 0.36; *P* < 0.0001), but not to such a degree as to preclude them from having an independent prognostic role, as shown by multivariate analyses.

**Figure 1:**
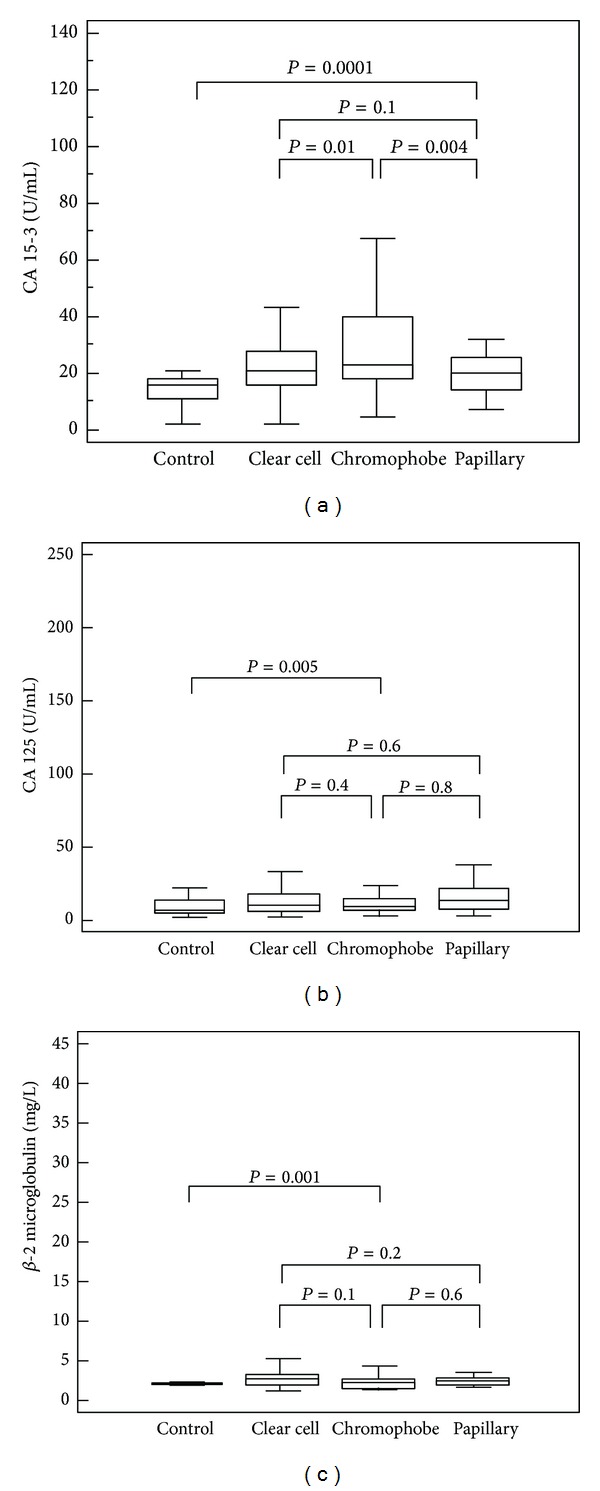
Differences in median CA 15-3 (a), CA 125 (b), and *β*-2 microglobulin (c) levels according to histological subtype and compared to healthy subjects (control).

Figure 1 illustrates the differences in median CA 15-3, CA 125, and *β*-2 microglobulin levels according to pathological subtype. The specificity and sensitivity of CA 15-3 for CSS and PFS are summarized in Table 4.

### 3.1. Cancer-Specific Survival

**Figure 2:**
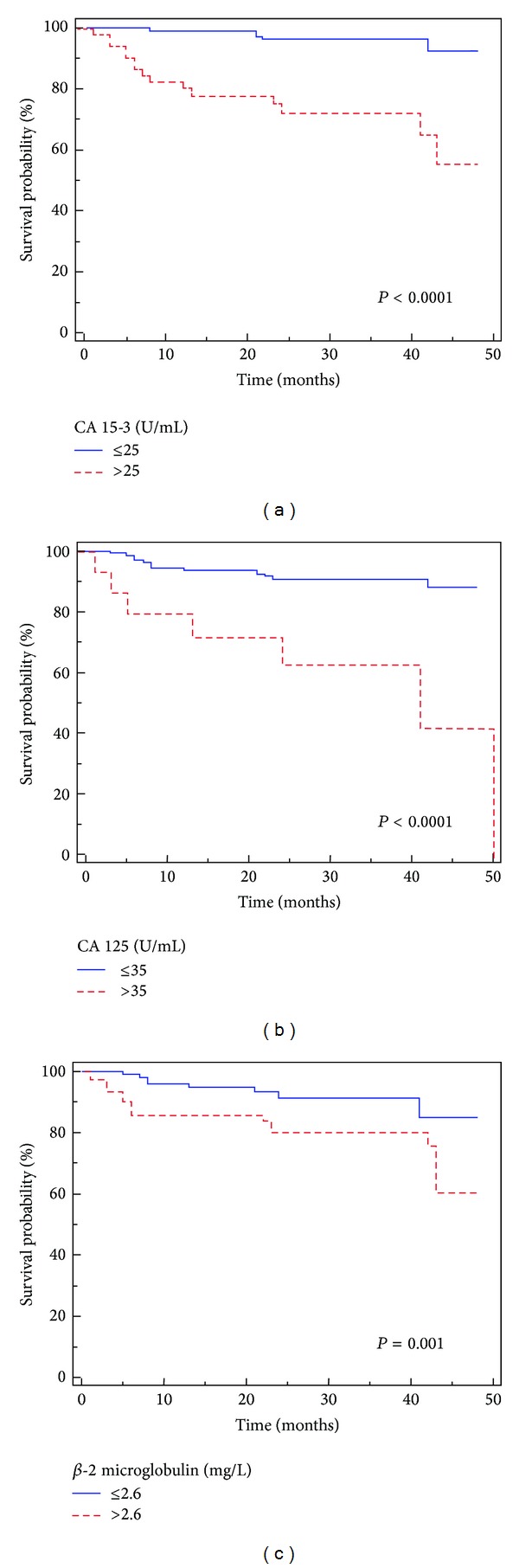
Kaplan-Meier cancer-specific survival (CSS) curves, stratified by CA 15-3 (a), CA 125 (b), and *β*-2 microglobulin (c) serum levels.

After a median followup of 23.4 months (95% CI: 21–26), 36 (10.8%) patients had died of RCC. Kaplan-Meier survival curves for CSS, stratified by the three biomarkers levels, are shown in Figure 2. CSS was significantly decreased for patients with high levels of CA 15-3, CA 125, and *β*-2-microglobulin (*P* < 0.0001, *P* < 0.0001, and *P* = 0.001, resp.). Univariate analyses for the predefined variables showed that age, pathological stage, presence of nodal and visceral metastases, Fuhrman grade, and high levels of CA 15-3, CA 125, and *β*-2-microglobulin were significantly associated with the risk of death (all *P* = 0.0001). At multivariate analysis by Cox regression modeling, age, the presence of visceral metastases, and high levels of CA 15-3 were independent adverse prognostic factors for CSS (Table 5(a)).

Subgroup analyses for CA 15-3, comparing localized (pT1-2, N0/M0) and advanced disease (pT3-4 and/or N+/M+) are shown in Figures 4(a) and 4(b).

### 3.2. Progression-Free Survival

**Figure 3:**
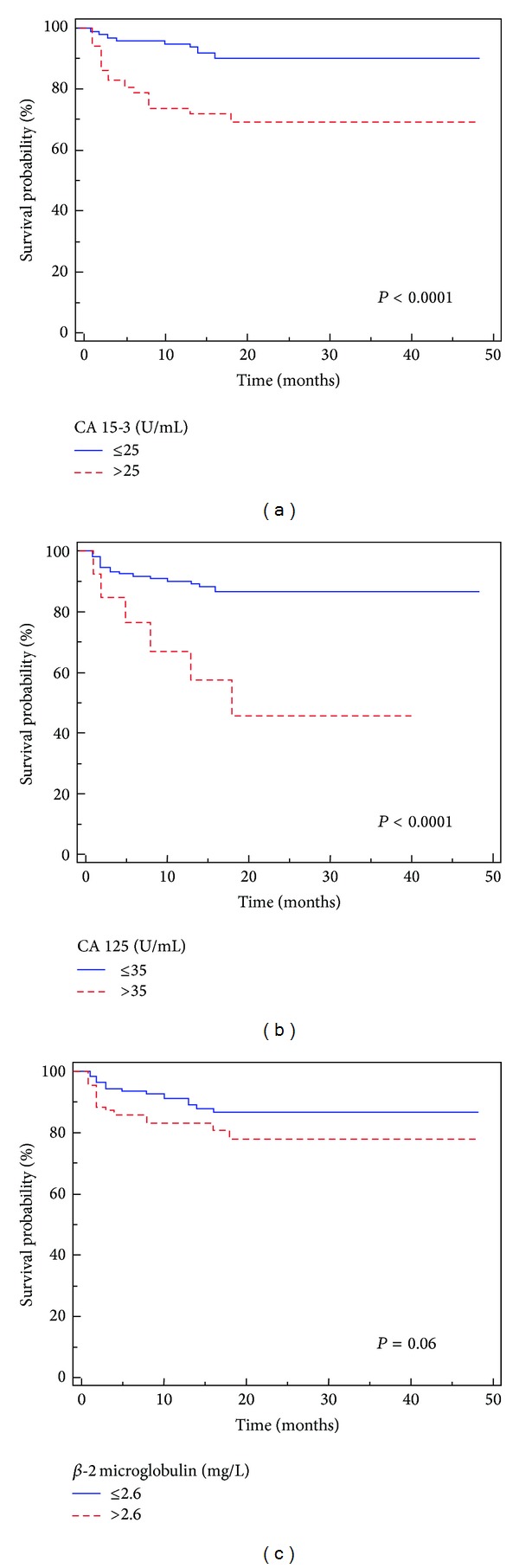
Kaplan-Meier progression-free survival (PFS) curves, stratified by CA 15-3 (a), CA 125 (b), and *β*-2 microglobulin (c) serum levels.

**Figure 4:**
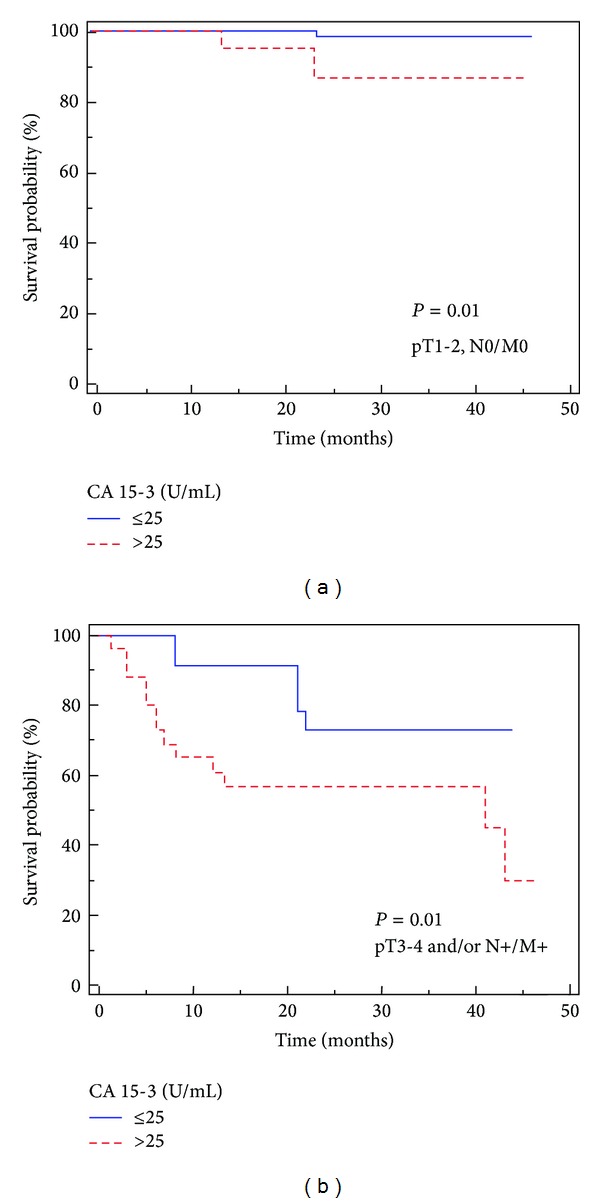
Kaplan-Meier cancer-specific survival (CSS) curves for organ-confined (a) and advanced RCC (b), plotted against pre-operative CA 15-3 values.

After surgery, 52 (15.6%) patients showed disease progression, after a median PFS of 21 months (95% CI: 17–24). Kaplan-Meier survival curves for PFS, stratified by the three biomarkers levels, are shown in Figure 3. The PFS was significantly decreased for patients with abnormal levels of CA 15-3 (*P* < 0.0001) and CA 125 (*P* < 0.0001) but not for *β*-2 microglobulin (*P* = 0.06) Univariate analyses for the predefined variables showed that pathological stage, presence of nodal and visceral metastases, Fuhrman grade, and high levels of CA 15-3 and CA 125 were significantly associated with the risk of death (all *P* = 0.0001). At multivariate analysis only pT stage, presence of nodal and visceral metastases, and high levels of CA 15-3 and CA 125 were independent adverse prognostic factors for PFS (Table 5(b)).

## 4. Discussion

The discovery and utilization of tumor markers have improved early detection, prognosis, and followup management of many malignancies. Optimal treatment and cure depends not only on accurate, early diagnosis but also on reliable followup to ensure an efficient detection of clinical recurrence. In some urologic tumors, prostate-specific antigen (PSA), *α*-fetoprotein, and *β*-human chorionic gonadotropin (*β*-hCG), together with new emerging biomarkers [12–14], are invaluable tools for the detection, staging, and monitoring of men diagnosed with prostate or testis cancer. Moreover, the availability of preoperative markers can contribute to select patients that could benefit from neoadjuvant treatment and aid clinical decision-making and choice of the best surgical option.

In this study we prospectively evaluated the role of three common serum biomarkers in a population of patients who underwent radical or partial nephrectomy for RCC. The biomarkers were measured in serum samples collected the day before surgery. Patients with all known clinical conditions that could cause abnormal values of these circulating proteins were excluded from the study.

CA 125 (also known as MUC16) is a high molecular weight glycoprotein used mainly as a diagnostic biomarker for ovarian cancer. Elevated levels of this protein have also been found in tumors of the pancreas, lung, breast, and bladder, as well as in different benign diseases.

Grankvist et al. [15] reported, for the first time, elevated levels of CA 125 in patients with RCC. CA 125 serum levels were particularly increased in patients with high grade tumors and advanced disease, and at multivariate analysis this biomarker resulted in an independent prognostic factor for overall survival.

An immunohistochemical study confirmed these findings and showed that patients with positive CA 125 tissue expression had a 2.5-fold increased risk of death from RCC than patients with negative staining [16]. This study also confirmed a positive association between CA 125 expression and tumor stage and grade, in accordance with the Grankvist et al. results [15]. In our study, only 9.6% of patients had increased serum levels of this marker, especially subjects with advanced disease. We also observed a positive correlation between CA 125 levels, tumor size, and Fuhrman grade. Kaplan-Maier curves showed clear differences in CSS and PFS between patients with values below and above 35 U/mL.

Beta-2 microglobulin is a component of major histocompatibility complex (MHC)/human leukocyte antigen (HLA) class I molecules, and high serum levels are generally present in hematological malignancies and in nonneoplastic renal and liver diseases. Downregulation of HLA class I molecules frequently occurs in many human cancers and is believed to be a mechanism of tumor escape from the immune surveillance system. Unlike in other tumors, RCC presents a low frequency of HLA class I alterations, in association with a higher expression of *β*-2 microglobulin. Previous studies have shown an increased *β*-2 microglobulin expression in renal cancer tissues and elevated serum levels in patients with RCC [17–19]. Moreover, *β*-2 microglobulin has been shown to promote proliferation, invasion, and migration of human RCC cells *in vitro* [20]; also, *β*-2 microglobulin-mediated signaling converges on PI3K/Akt, ERK, and JNK pathways to sustain cell growth and RCC cell survival [21].

In our findings, *β*-2 microglobulin levels were increased in about 30% of patients, above all in subjects with metastatic disease. A significant correlation resulted between serum *β*-2 microglobulin values and tumor stage and grade. Kaplan-Meier survival curves stratified by *β*-2 microglobulin levels demonstrated that CSS was significantly decreased in patients with values above 2.6 mg/dL.

CA 15-3 (also known as MUC1 or EMA) is the serum marker most widely used in breast cancer. Like CA 125, MUC1/CA 15-3 is a member of the mucin family and is a membrane-associated O-glycoprotein with a large extracellular domain. CA 15-3 is expressed on the apical membrane of almost all glandular epithelia, and in normal kidney it is localized in the distal convoluted tubules and in the collecting ducts [22]. CA 15-3 has a role in cell adhesion and cellular polarity and through its intracellular domain is implicated in signal transduction, interacting with the EGF receptor, and activating the mitogen-activated protein (MAP) kinase pathway [23]. Previous immunohistochemical studies have shown that CA 15-3 is upregulated in RCC and that overexpression is associated with a higher tumor grade and stage [24–27]. Aubert et al. [28] provided evidence, for the first time, that hypoxia induces MUC1 in renal cancer cells and showed that this up-regulation was controlled by the transcriptional activity of HIF1*α*. Moreover, a higher MUC1 expression was shown to be correlated with the metastatic status of patients. Grankvist et al. [15] reported elevated serum levels of CA 15-3 in up to 30% of patients in the most advanced clinical stage and grade but failed to identified this tumor marker as an independent prognostic factor for survival at multivariate analysis. Similarly, Briasoulis et al. reported abnormal serum levels of this protein in 23% of RCC patients with metastatic disease [29]. Therefore, the prognostic relevance of CA 15-3 is still under debate, since data demonstrating a role on patients' outcome and RCC biology are not yet fully established.

Our multivariate analysis showed that high levels of CA 15-3, together with the presence of visceral metastases, were significantly predictive of risk of death. Moreover, this biomarker remained an independent prognosticator of outcome for PFS. Kaplan-Meier curves showed clear differences in CSS and PFS between patients with CA 15-3 values below and above the normal values. Moreover, subgroup analyses comparing localized (pT1-2, N0/M0) and advanced disease (pT3-4 and/or N+/M+) confirmed significant differences in CSS for CA 15-3 values ≤25 or >25 U/mL in both clinical settings. Stratifying patients by histological subtypes, we found significantly higher levels of CA 15-3 in chromophobe RCC; these findings are in accordance with the Langner et al. [27] immunohistochemical analyses.

Among the biomarkers analyzed, CA 15-3 seems to be a new potential therapeutic target for patients with RCC, considering that this protein affects the invasive behavior of renal cancer cells and is directly regulated by HIF1*α*, which is the main signaling pathway in RCC carcinogenesis.

## 5. Conclusions

Many experimental studies have shown that CA 15-3, CA 125, and *β*-2 microglobulin play important roles in the migration and invasion properties of renal cancer cells, but the utility of these biomarkers in clinical settings is controversial. We found that CA 15-3, CA 125, and *β*-2 microglobulin are preoperatively increased in some patients. Elevated serum levels of CA 15-3 were correlated to the clinical stage and tumor grade. Moreover, CSS and PFS were significantly shorter for patients with elevated CA 15-3 levels than for those with normal CA 15-3 values. Further prospective studies based on larger series are warranted to confirm the utility of this biomarker.

## Figures and Tables

**Table 1:** Patients clinical and pathological characteristics.

Characteristic	RCC patients (*n* = 332)	Healthy subjects (*n* = 113)	*P* value
Age (years)			
Median	63	61	
Range	20–85	22–84	
Gender			
M	230 (69.3%)	81 (71.6%)	
F	102 (30.7%)	32 (28.4%)	
Type of nephrectomy			
Radical	167 (50.3%)		
Partial	165 (49.7%)		
Tumor size (cm)			
Median	4.5		
Range	0.4–24		
Pathological stage			
pT1a	141 (42.5%)		
pT1b	84 (25.3%)		
pT2	28 (8.4%)		
pT3	79 (23.8%)		
LN metastases	21 (6.3%)		
Visceral metastases	27 (8.1%)		
Fuhrman grade			
G1-2	212 (63.8%)		
G3-4	120 (36.2%)		
Histological subtype			
Clear cell	246 (74.1%)		
Papillary	32 (9.6%)		
Chromophobe	54 (16.3%)		
CA 15-3 (U/mL)			
Median	21.74	16.02	<0.0001
95% CI	20.05–22.61	14.44–16.56	
CA 125 (U/mL)			
Median	10.56	7.98	<0.0001
95% CI	9.61–12.00	7.03–9.48	
*β*-2 m (mg/L)			
Median	2.00	1.70	<0.0001
95% CI	1.90–2.10	1.50–1.72	

**Table:** (a)

	Tumor size				*P* value
	≤7 cm		>7 cm		
	Median	95% CI	Median	95% CI
CA 15-3 (U/mL)	19.79	18.72–21.20	32.84	28.90–37.10	<0.0001
CA 125 (U/mL)	10.02	9.41–11.31	14.75	10.62–20.77	0.002
*β*-2 m (mg/L)	1.90	1.80–2.00	2.30	2.10–2.70	<0.0001

**Table:** (b)

	Fuhrman grade				P value
	G1-2		G3-4		
	Median	95% CI	Median	95% CI
CA 15-3 (U/mL)	20.06	18.99–21.87	25.39	22.11–27.85	0.0007
CA 125 (U/mL)	9.67	9.16–11.01	13.89	10.49–16.17	0.002
*β*-2 m (mg/L)	2.00	1.80–2.00	2.31	1.99–2.60	0.001

**Table:** (a)

	LN metastases				*P* value
	N0		N+		
	Median	95% CI	Median	95% CI
CA 15-3 (U/mL)	20.79	19.71–22.30	47.27	31.15–67.73	<0.0001
CA 125 (U/mL)	10.36	9.48–11.56	17.60	9.05–104.90	0.001
*β*-2 m (mg/L)	2.00	1.90–2.10	2.70	1.80–5.90	0.002

**Table:** (b)

	Visceral metastases				*P* value
	M0		M+		
	Median	95% CI	Median	95% CI
CA 15-3 (U/mL)	20.22	18.72–21.93	32.23	27.05–52.35	<0.0001
CA 125 (U/mL)	9.63	8.33–11.00	22.15	10.80–94.00	<0.0001
*β*-2 m (mg/L)	1.85	1.78–2.00	2.30	1.93–2.60	0.01

**Table 4:** Sensitivity and specificity of CA 15-3 for cancer-specific survival (CSS) and progression-free survival.

	Sensitivity	95% CI	Specificity	95% CI
CSS	83.3%	67.2–93.6	75.3%	69.8–80.3
PFS	63.4%	49.0–76.4	75.6%	69.9–80.7

**Table 5:** Multivariate analysis for CSS (a) and PFS (b).

Variable	*P* value	HR	95% CI
(a) CSS			
Age	0.03	0.95	0.90–0.99
T	0.76		
N	0.21		
M	0.0001	2.91	1.60–4.32
Fuhrman grade	0.06		
CA 15-3	0.002	1.05	1.02–1.08
CA 125	0.11		
*β*-2 microglobulin	0.97		

(b) PFS			
Age	0.53		
T	0.001	3.09	1.95–4.91
N	0.03	3.36	1.06–10.60
M	0.0001	2.06	1.01–3.19
Fuhrman grade	0.71		
CA 15-3	0.003	1.04	1.01–1.07
CA 125	0.007	0.96	0.92–0.99
*β*-2 microglobulin	0.51		
